# Trophically integrated ecometric models as tools for demonstrating spatial and temporal functional changes in mammal communities

**DOI:** 10.1073/pnas.2201947120

**Published:** 2023-02-06

**Authors:** Rachel A. Short, Jenny L. McGuire, P. David Polly, A. Michelle Lawing

**Affiliations:** ^a^Department of Natural Resource Management, South Dakota State University, Rapid City, SD 57703; ^b^Department of Ecology and Conservation Biology, Texas A&M University, College Station, TX 77843; ^c^School of Biological Sciences, Georgia Institute of Technology, Atlanta, GA 30332; ^d^School of Earth and Atmospheric Sciences, Georgia Institute of Technology, Atlanta, GA 30332; ^e^Interdisciplinary Graduate Program in Quantitative Biosciences, Georgia Institute of Technology, Atlanta, GA 30332; ^f^Department of Earth and Atmospheric Sciences, Indiana University, Bloomington, IN 47405

**Keywords:** ecometrics, Artiodactyla, Carnivora, locomotion, vegetation cover

## Abstract

We are in a modern biodiversity crisis that will restructure community compositions and ecological functions globally. Large mammals, important contributors to ecosystem function, have been affected directly by purposeful extermination and indirectly by climate and land-use changes, yet functional turnover is rarely assessed on a global scale using metrics based on functional traits. Using ecometrics, the study of functional trait distributions and functional turnover, we examine the relationship between vegetation cover and locomotor traits for artiodactyl and carnivoran communities. We show that the ability to detect a functional relationship is strengthened when locomotor traits of both primary consumers (artiodactyls, n = 157 species) and secondary consumers (carnivorans, n = 138 species) are combined into one trophically integrated ecometric model. Overall, locomotor traits of 81% of communities accurately estimate vegetation cover, establishing the advantage of trophically integrated ecometric models over single-group models (58 to 65% correct). We develop an innovative approach within the ecometrics framework, using ecometric anomalies to evaluate mismatches in model estimates and observed values and provide more nuance for understanding relationships between functional traits and vegetation cover. We apply our integrated model to five paleontological sites to illustrate mismatches in the past and today and to demonstrate the utility of the model for paleovegetation interpretations. Observed changes in community traits and their associated vegetations across space and over time demonstrate the strong, rapid effect of environmental filtering on community traits. Ultimately, our trophically integrated ecometric model captures the cascading interactions between taxa, traits, and changing environments.

## Significance

Mammals live in habitats compatible with their locomotor traits. We show that the functional relationship between traits and the environment can be a tool for reconstructing ancient vegetation cover and identifying areas of mismatch between traits and land cover. We demonstrate that these tools are stronger with a more completely sampled community, examining models of species at both the primary consumer (herbivore) and secondary consumer (carnivore) trophic levels. Metrics for evaluating trait-environment relationships over time have the potential to inform strategies for restoring functional relationships in ecosystems and prioritizing functionally intact areas for conservation.

Climate and habitats are changing at a rapid pace (1–3), shifting species ranges and resulting in a redistribution of plants and animals in Earth’s ecosystems (4–7). Relationships between functional traits and the environmental conditions in which they occur form the foundation of functional ecosystems (8–12). Ecometric methods facilitate comparisons of communities based on the distribution of functional traits both within and between communities in relation to their environment, even if they share no species in common (13–15). Ecometric relationships have been used to study rates and magnitudes of biotic responses to environmental changes, measure ecological function in communities past and present, and reconstruct paleoenvironments (14–16). Examples include using leaf margins of deciduous woody plants for paleotemperature (17, 18), molar crown height of large mammalian herbivores for ecosystem aridity and precipitation (19–21), and reptile body size for paleotemperature (22). These tools have allowed investigations of the environmental filtering processes that have sorted functional traits into the community distributions that we find today (16, 23–25). Ecometric approaches enhance our ability to integrate data sampled at different spatial and temporal scales, allowing us to understand the dynamics of ecosystem function at local, regional, and global areas over decades, centuries, and millennia (10, 14, 15).

Large mammals play an important role in ecosystem function (26–28) and are known to be sensitive indicators of ecosystem change. Here, we study two groups that occupy different trophic levels, Artiodactyla (bison, deer, giraffes, and their relatives), which are almost exclusively primary consumers (herbivores), and Carnivora (cats, wolves, bears, and their relatives), most of which are secondary consumers (carnivores and omnivores). Herbivores are closely tied to the climate because it directly affects the habitats to which they are adapted, the species that are their predators, and the vegetation they consume (29–34). Carnivores and omnivores are closely tied to their prey species, some of which are artiodactyls, the habitats to which they are adapted, and the species with whom they compete (35, 36). Both groups contribute to ecosystem function through biomass consumption, nutrient cycling, energy flow, fire modification, and species interactions (26–28, 37–39). All trophic levels are susceptible to climate change, exploitation, habitat fragmentation, agricultural expansion, deforestation, and urbanization (27, 40–43), threats that disproportionately impact large herbivores and carnivores (44–46).

**Fig. 1.**
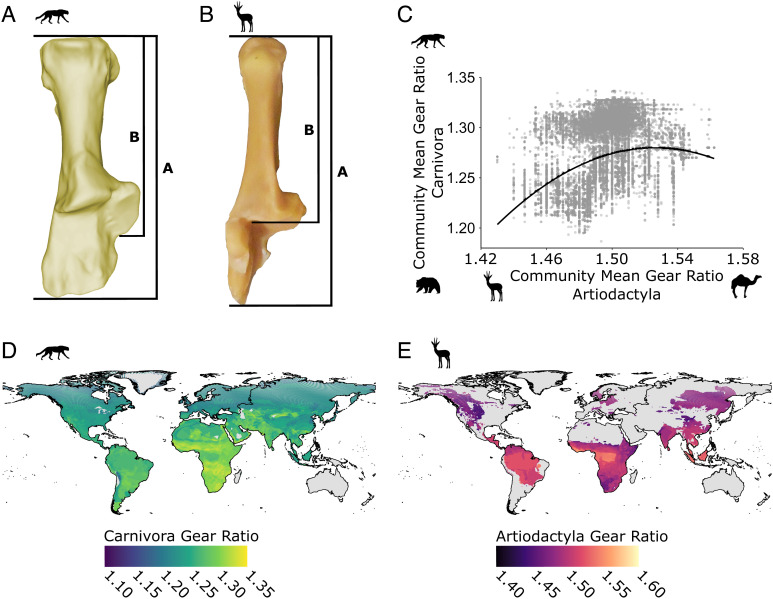
Ankle gear ratio measures and geography. (*A*) Carnivoran calcaneum (*Canis familiaris*) and measurement schema. (*B*) Artiodactyl calcaneum (*Odocoileus virginianus*) and measurement schema. In (*A*) and (*B*), measurement A is the total length of the calcaneum and measurement B is the length of the calcaneal tuber to the sustentacular process. Gear ratio is calculated as measurement A divided by measurement B following Polly (36) and Short and Lawing (37). (*C*) Global relationship between the ankle gear ratios of carnivorans and artiodactyls (y = −0.77x2 + 1.60x + 1.27, R^2^ = 0.14, *P* < 0.001). (*D*) Global distribution of carnivoran ankle gear ratio. (*E*) Global distribution of artiodactyl ankle gear ratio. Silhouettes are from PhyloPic.org.

We use locomotor traits of artiodactyls and carnivorans to assess whether using taxa from across trophic levels influences ecometric results and armed with that knowledge, whether trait-environment relationships have shifted through time at five paleontological sites. One straightforward measure of locomotor performance is the ratio of the distal to proximal hind limb segments, which relates to stance and stride (47–56). A proxy for this ratio is the ankle gear ratio, which is measured from a single bone, the calcaneum (Fig. 1 *A* and *B*), and can be measured equally well in living mammals and in fragmentary fossils. Note that the ankle gear ratio is positively correlated with distal limb length and speed in carnivorans but negatively correlated in artiodactyls (48). In Carnivora, a high ratio (e.g., as found in cats) arises from elongation of the distal portion of the calcaneum and results in a more digitigrade posture, longer strides, and greater cursoriality or springing ability than in carnivorans with low ratios (e.g., bears) (16, 47). In Artiodactyla, a longer distal foot, longer strides, and greater cursoriality are associated with a lower calcaneal ratio because their highly derived astragalus forms an extra segment, and the proximal end of the calcaneum envelops and supports its upper joint. The distal portion of the calcaneum is therefore longer in taxa with low ratios (e.g., hippos and camels), allowing for a greater range of rotation during limb extension, and shorter in more cursorial taxa (e.g., gazelles) (48, 57).

Functionally, the ankle gear ratio captures the trade-off between speed and strength of forward propulsion and is directly related to efficiency of running, walking, springing, climbing, and digging (58). The ankle ratio thus reflects the efficiency with which large mammals navigate landscapes like brush, forest, or open areas (16, 47, 48). The distribution of the ankle ratio within and among communities is correlated with the average openness of the environment, and the ratio’s variance is correlated with local variety in vegetative cover (47–56). If either the vegetation cover or the community composition is altered, the relationship becomes mismatched until the functional alignment of the fauna and environment can become realigned either by restoration of the vegetation or replacement of the fauna with new species whose traits match the altered landscape. Ecometric models can measure the alignment and mismatch of traits and their environments. Projected though time, ecometric models can provide new lines of evidence to contribute to paleoenvironmental interpretations and evaluating ecometric trait change through time will improve our understanding of past functional trait turnover.

Here, we construct a likelihood model of the relationship between vegetation cover and the community-level means and variances of the ankle gear ratios (an ecometric model). We use it to assess the geographic distribution of present-day functional traits and their relationship to vegetation cover at a global scale. We test whether ecometric models with multiple trophic levels are more informative than those including only single trophic levels. We also investigate how an ecometric likelihood model can provide more nuance to vegetation reconstructions and how they can be used to reconstruct past vegetation cover. We address three questions: a) How is gear ratio related between trophic levels and across geographic space? b) Does integrating primary consumers (artiodactyls) and secondary consumers (carnivorans) into a single ecometric model improve our estimates of vegetation cover? c) What can ecometric likelihoods tell us about trait-environment relationships across space and through time?

## Results

### Gear Ratios in Carnivorans and Artiodactyls Vary with Vegetation Type and between Continents.

Geographic variation in mean ankle gear ratio, and therefore locomotor function, is apparent on a global scale in both carnivoran and artiodactyl communities (Fig. 1). SD also varies significantly across vegetation types but has a lower percent explained by vegetation type than does the mean (*SI Appendix*, Table S1). Carnivoran ankle gear ratio is high in most of sub-Saharan Africa and southern Asia and low to medium across the Holarctic, whereas the artiodactyl ankle gear ratio is high at tropical latitudes and low to medium in temperate regions (Fig. 1 *D* and *E*). While the artiodactyls have greater ranges of SD in all vegetation types, except arctic, carnivorans have tighter, more consistent ranges of SD across vegetation types (*SI Appendix*, Figs. S1 and S2). Communities of artiodactyls display more variation in mean and SD of the ankle gear ratio in Africa than on any other continent, whereas communities of carnivorans display more variation of mean and SD in Asia (Fig. 1 *D* and *E* and *SI Appendix*, Table S2).

The relationship between mean gear ratios of carnivoran and artiodactyl communities is complex, exhibiting a positive relationship up to an inflection point (at artiodactyl mean gear ratio = 1.53), followed by a slight negative relationship. This relationship is best described by the second-order polynomial: y = −0.77x^2^ + 1.60x + 1.27, R^2^ = 0.14, *P* < 0.001 (Fig. 1*C*). A polynomial relationship is also seen in desert, evergreen forest, and grassland vegetation types (*SI Appendix*, Figs. S2 and S3). The relative relationship of the mean gear ratios is different in deciduous forests, where there is a positive, nearly linear relationship, between the two orders. In the few arctic areas in our study, both groups have consistently low gear ratio values (*SI Appendix*, Fig. S3).

There is a complex pattern of mean gear ratios for communities at the continent level. In Africa, where mean gear ratios for carnivoran communities are consistently high (canids and felids), mean gear ratios of artiodactyl communities are highly variable (*SI Appendix*, Fig. S4). There is a positive relationship between mean gear ratios of communities within Asia and North America, where felids, mustelids, and ursids co-occur in many forested environments and are replaced by canids in more open environments (23), but where most of the artiodactyls are more extreme cursors like cervids and antilocaprids. South America has a nearly flat relationship with carnivorans consistent and artiodactyls more variable. Europe has an inverse relationship that indicates more plantigrade carnivorans co-existing with the least cursorial artiodactyls (*SI Appendix*, Fig. S4 and Table S3).

### Trophically Integrated Communities Improve Our Understanding of Ecometric Relationships.

Three ecometric likelihood models were constructed to map vegetation cover (59, 60) onto the means and SDs of ankle ratios: a) carnivorans only, b) artiodactyls only, and c) trophically integrated artiodactyls and carnivorans. These models are used to calculate the likelihood of observing each vegetation type given the distribution of ankle ratios in the large mammal community [(14, 15); see also further description below]. When the functional relationship between vegetation and ankle ratio is strong, the models will estimate a single vegetation type with strong likelihood for each combination of mean and SD in ankle ratio. However, when the trait-environment relationship is weak, there will be more than one vegetation type with similar likelihoods, increasing the chances of an incorrectly estimated vegetation (14, 15). To evaluate each model, we calculated Cohen’s kappa (κ), which describes the proportion of correct estimates and ranges between 0 (no greater correct estimates than by chance) and 1 (perfect estimation) (15, 61, 62). This provides a measure of the strength of the functional relationship between traits and environment.

Trophically integrated ecometric models were better estimators of vegetation cover than either trophic level independently. Vegetation cover estimates from the trophically integrated model were correct for 80.8% of the geographic sampling points (n = 20,763, κ = 0.73, *P* < 0.001, *SI Appendix*, Table S4). Estimates based on carnivorans alone were accurate for only 57.7% of the sampling points (n = 47,270, κ = 0.45, *P* < 0.001, *SI Appendix*, Table S4) and those based on artiodactyls were accurate for 64.8% sampling points (n = 20,766, κ = 0.50, *P* < 0.001, *SI Appendix*, Table S4). We parsed the results of the integrated model by vegetation type and found that most vegetation estimates were correct in 77 to 87% of locations (*SI Appendix*, Table S4). The arctic was the exception with only 22% correct estimates (*SI Appendix*, Table S4). Only a small proportion of our data fall within the arctic vegetation category, and the gear ratios of carnivorans and artiodactyls that live there do not differ substantially from other vegetation types.

### Ecometric Likelihood Models Provide a More Nuanced Understanding of Ecometric Estimates.

Ecometric spaces show how the frequency of each vegetation type is spread across the community-level trait space and can be used to visualize the trait-environment relationship [*SI Appendix*, Fig. S5; (15)]. These ecometric spaces allow the likelihood of a vegetation type to be estimated for any combination of mean and variance of gear ratios by tabulating the frequency of vegetation types that occur within each trait combination. The ecometric space has been divided into an arbitrary but small number of bins and normalized by the total number of communities. Individual vegetation types tend to be associated with quite specific and narrow combinations of traits in carnivoran communities but with a wider variety of trait distributions in artiodactyls and in the trophically integrated model.

**Fig. 2.**
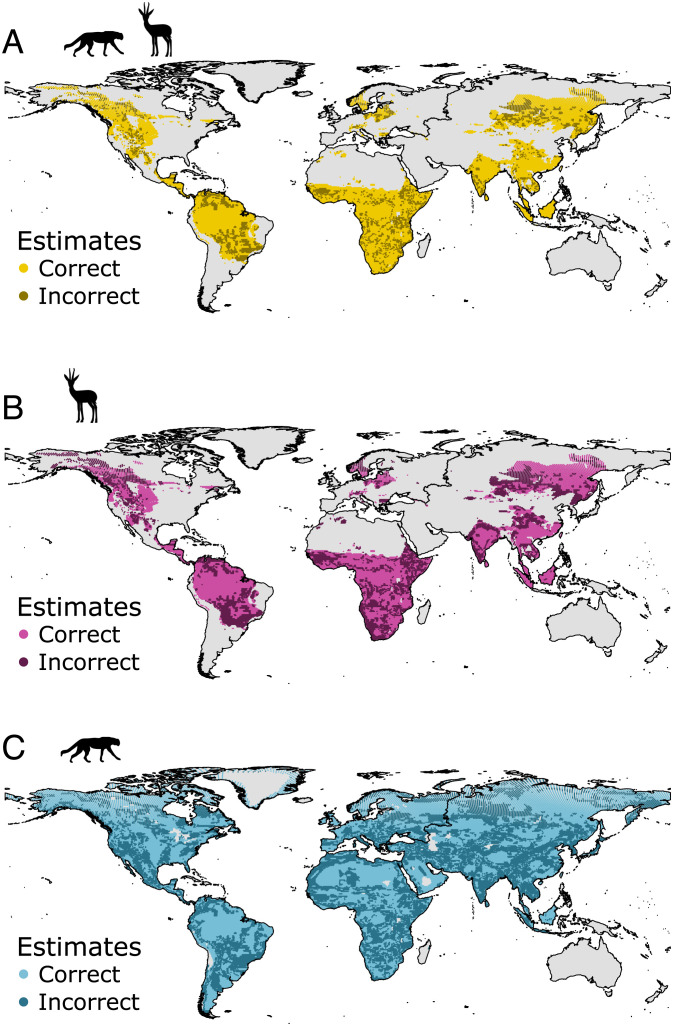
Spatial patterns of correct and incorrect trait-based vegetation type predicted from three ecometric models. (*A*) Trophically integrated ecometric model. (*B*) Artiodactyl ecometric model. (*C*) Carnivoran ecometric model. Silhouettes are from PhyloPic.org.

We used the likelihoods to calculate an “ecometric anomaly” to compare the accuracies of our three ecometric models. As observed above, the most likely vegetation type estimated from a model is not always correct, and a more nuanced measure of its accuracy is how close the likelihood of the correct vegetation type is to the one estimated by the model (*SI Appendix*, Fig. S6). The model is more nearly correct if the true vegetation has a high likelihood rather than a low one. Thus, we calculated the ecometric anomaly as the likelihood of the most likely vegetation type minus the likelihood of the observed vegetation type. The ecometric anomaly ranges from 0 when the predicted vegetation is correct to 1 when the correct vegetation has a zero likelihood under the model. All three ecometric models were skewed toward 0, meaning that the correct vegetation type almost always had a high likelihood regardless of whether it was the highest (*SI Appendix*, Fig. S7 and Table S4). The skew toward correctness was greatest for the trophically integrated model and least in the artiodactyl model (Fig. 2) (*SI Appendix*, Figs. S7 and S8 and Table S4).

### Application of Ecometric Likelihood Models to Five Paleontological Sites.

**Fig. 3.**
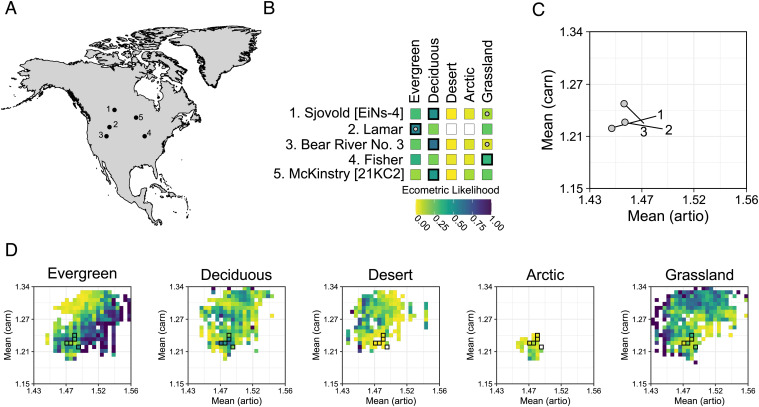
Ecometric analysis of five paleontological sites in North America. (*A*) Geographic locations of sites. (*B*) Likelihoods of each vegetation type for each site. A darker likelihood (closer to 1) indicates a higher likelihood of that vegetation type given the vegetation types of the communities that occur within that trait bin in the trophically integrated ecometric model. Bold boxes indicate the most likely paleovegetation based on fossil traits and the gray dots indicate the most likely modern vegetation based on the modern traits at the same locations. Two sites (Fisher and McKinstry) do not have modern trait values. (*C*) Vectors of trait change at three of the paleontological sites show the direction and magnitude of change from the time of deposition (no dot) to the modern (dot). (*D*) Ecometric spaces show the distribution of the likelihood surfaces for all trait bins and the black hollow boxes represent the location of the five paleontological sites in ecometric space.

The ecometric likelihoods illustrate how the integrated model can be used to understand changes in traits and vegetation estimates at five Holocene sites in North America (Fig. 3 and *SI Appendix*, Table S5). Vegetation data from previous paleoenvironmental studies of each site were used for comparison to ecometric reconstructions that assigned each site a vegetation type from our schema. At the same locations of the fossil sites, we also compared vegetation data estimated from the modern communities to the vegetation observed in Matthews’ dataset (*SI Appendix*, Table S6). Two sites (Fisher and McKinstry) did not have corresponding modern communities for comparison because they exist in an area of North America that is depauperate of modern artiodactyls. Fisher paleovegetation was interpreted as tundra and boreal forest (63–65). The ecometric model weakly supports evergreen and grassland with similar probabilities (Fig. 3*B*). McKinstry was interpreted as a mixed forest of evergreens and deciduous (66, 67); grassland had the highest probability from the ecometric model (Fig. 3*B*).

Prior studies interpreted the Lamar site as having a mixed mosaic of vegetation types, including evergreen-dominated forest and grasslands, previously dense tall grass, and now sparse and arid grass (68, 69). Modern observed vegetation from Matthews’ dataset, ecometric estimations of modern estimated vegetation (Fig. 3*B*), and ecometric estimations of past vegetation all indicate evergreen (Fig. 3*B*), with a higher likelihood in the past. Prior literature interpreted Bear River No. 3, which is in the Great Salt Lake Basin of Utah, to be a mosaic of habitats, including grassland with increasing pockets of deciduous vegetation (70–72). The ecometric model estimated deciduous vegetation for the fossil site with a very high likelihood (Fig. 3*B*). There is a mismatch between the modern observed vegetation (grassland) and the modern estimated vegetation (deciduous), though this could be because of the increasing mosaic landscape (70–72). At Sjovold, there is a mismatch between the paleovegetation interpretation of grassland to parkland transition (73–77) and the ecometric estimate of moderately high likelihoods of both deciduous and evergreen (Fig. 3*B*), possibly because of the large age range of the site (0 to 4,500 y). The ecometric model does correctly estimate modern grassland vegetation at Sjovold (Fig. 3*B*).

Community trait values from the paleontological record were compared to modern community trait values to demonstrate how ecometric methods can be used to evaluate functional trait turnover through time (Fig. 3 *A*–*C*). The three sites with trait values for both time periods demonstrated consistent trait shifts toward lower artiodactyl mean gear ratios through time when plotted in ecometric space (Fig. 3*C*). This is largely because of the replacement of bovids (*Bison* and *Ovis*) and large-bodied cervids (*Alces* and *Cervus*) with *Odocoileus* (*SI Appendix*, Table S7). The shifts in trait values were associated with correct estimates of modern vegetation at all three sites, suggesting that this faunal turnover was associated with vegetation change (*SI Appendix*, Table S6).

## Discussion

We present an ecometric model that integrates two trophic levels. Although both trophic groups demonstrate strong trait-environment correlations individually, our trophically integrated model is better at estimating vegetation cover from traits than either alone. This suggests that the two taxonomic orders are contributing unique information to more accurately detect the functional relationship with vegetation. Carnivorans are adapted to move across the landscape in a variety of ways (e.g., scansorial, arboreal, and cursorial), and artiodactyls are primarily cursorial but with slight variations (e.g., stotting versus trotting). These functional properties contribute to the sorting of species into different communities in different vegetation types which are reflected in the differences in trait means and SDs (*SI Appendix*, Figs. S2 and S3). Generally, communities of carnivoran species have a greater variety of locomotor strategies contributing to the larger ranges of mean gear ratios and tighter ranges of higher SDs across vegetation types. Communities of artiodactyls have a more constrained, cursorial locomotor strategy producing smaller ranges of mean gear ratios, especially in deciduous and grassland vegetation, and larger ranges of smaller SDs. Yet, at the continental scale, artiodactyls demonstrate greater ranges of gear ratios than carnivorans in Africa, North America, and South America; the two orders are more similar in Asia and Europe (*SI Appendix*, Fig. S4).

Our models provide a way to use locomotor strategy and its relationship with vegetation cover to assess ecosystem change over time. Locomotion strategies are only functionally successful when they are suited for the environment in which the animal lives. When either the trait or the environment is changed, a mismatch in the functional relationship occurs. This mismatch can act as an assessment tool either to identify where the mismatch occurs when the model produces an incorrect estimate or as a nuanced measure of degree of mismatch when the ecometric anomaly is used. Ecometric models can refine interpretations of paleoenvironment by considering the likelihood of each vegetation type. The ecometric anomaly can also provide more detailed information for the communities and trait bins. For instance, in a community with a low ecometric anomaly, the vegetation estimate may have been almost estimated correctly because its likelihood was nearly as high as the maximum likelihood. However, the occurrence of two nearly equal likelihoods suggests low power within that trait bin. Further analyses of the ecometric likelihoods and their anomalies will enhance our understanding of biodiversity change across spatial and temporal scales, supporting the development of long-term, geospatial knowledge of ecosystem function (10, 14, 15).

Using ecometric likelihood models, our paleovegetation reconstructions can confirm and refine prior interpretations of paleontological sites. North American vegetation and associated climates have shifted across the Holocene (7), driving community-level traits to rapidly reconfigure as a result of environmental filtering. When working with paleontological sites, there may be time-averaging that results in species from different times being included in the same community. However, we minimize this issue by limiting the sites that we include to those that span 5,000 y or less. Even when there are mismatches or low likelihoods, the models provide information about how confidently we can interpret vegetation cover, and a mismatch might be expected in a mosaic habitat where animals are traversing diverse vegetation, suggesting that not all mismatches are completely incorrect. Additionally, the Fisher site only has low to moderate likelihoods across vegetation types, indicating that its particular trait combination is not well differentiated among vegetation types. Plotting the community-level functional traits through time within an ecometric space shows shifting of traits occurring between the past and modern communities (Fig. 3*C*). This pattern has also been documented in the body size and gear ratios of historical and modern communities in Kenya (48, 78), suggesting that faunal communities have been undergoing a functional shift in the Holocene.

Whether the degree of mismatch between locomotor traits and vegetation can be used as a measure of functional disruption of an ecosystem, especially anthropogenically driven disruption, depends on how quickly trait distributions reach equilibrium relative to the rate of landscape modification. Results presented here and in previous studies (24, 25) indicate that a general equilibrium is maintained between locomotor traits and vegetation type because the functional relationship is strong. Further, because organisms cannot persist in environments that are incompatible with their traits, mismatches should reach equilibrium. We see this at the sites where the integrated model correctly estimated changes from the past to the modern vegetation. Even the Pleistocene megafaunal extinction, which removed many large-bodied species from mammal communities on most continents, did not cause a large or persistent trait mismatch (79–82). Ecometric trait distributions tracked the vegetational and climatic changes of glacial–interglacial cycles which occurred rapidly compared to the timescales of trait evolution (15, 23, 83–87).

But, while rapid on an evolutionary scale, those sorting processes may or may not have lags following the vegetation changes that drove them. Carnivoran locomotor trait composition in the American Midwest was not appreciably different in the dense deciduous forests of the pre-European 18th century and the Sangamonian forests of the last interglacial period. Even though the Sangamonian communities included extinct Dire wolves (*Canis dirus*) and saber-toothed cats (*Smilodon fatalis*) (15), the trait compositions changed in association with the vegetation, leading to the conclusion that the similarity between the two time periods was due to trait-environment sorting. Between 1,800 and the present in the Midwest, carnivore locomotor trait composition changed almost as much in response to 19th-century deforestation as it did in response to the last deglaciation, including the megafaunal extinction (15). Yet, the 19th-century Midwestern carnivoran communities lost some trait diversity, as indicated by smaller SDs, largely because of the extirpation of low-ratio bears and high-ratio wolves and mountain lions; though the community trait mean did not change (15). During this time, dense forests were transformed into open agricultural “prairies” and the static means in combination with changing SDs produced an entire region of “nonanalog” trait compositions not found anywhere else in North America (15). This suggests that trait-environment mismatches may indeed persist for decades or even a century and, thus, can be a valuable metric for ecosystem function.

Though many functional traits are strongly correlated with phylogeny, the mechanism of trait sorting depends on the performance of the trait regardless of its phylogenetic history. The ecometric trait distribution in a community is not itself a property of any one phylogenetic branch tip but a distribution of tip values from many points on a phylogeny. Thus, one cannot and should not apply phylogenetic corrections to ecometric means and SDs. For example, in carnivorans, felids and canids have the highest gear ratios, mustelids and mephitids have intermediate ones, and procyonids and ursids have the lowest yielding phylogenetic correlations measured with Blomberg’s *K* as high as 0.58 (23). This is similar to artiodactyls with hippopotamids and camelids at the high end of gear ratios and antilocaprids, bovids, and cervids at the low end (48). Community compositions that arise from locomotor trait sorting of these species also have a substantial phylogenetic structure because similar trait values mean that closely related species are independently sorted into the same habitats. For example, in North America, boreal forests are phylogenetically dominated by mustelids, open basin-and-range country by canids, and neotropical forests by felids and procyonids (23). For an ecometric correlation to arise from phylogeny, all members of a community in one environment would have to be each other’s closest relatives compared to the members of a community in another environment. But with 82% of the communities sampled in North America including distantly related canids and felids, this is demonstrably not the case. Additionally, community compositions have been restructured over geologically short intervals of thousands to tens of thousands of years, such as at the last deglaciation (79–81). Conversely, phylogenetic patterns of locomotor traits arose over tens of millions of years, a difference of four to five orders of magnitude (16, 23). Finally, the efficiency of one species’ ankle gear ratio does not depend on the ratios of its closest relatives, only on the interaction between its functionality and the local environment. Clades can face geographic barriers that prevent them from dispersing to areas where their traits would perform well (11, 15–16). For example, hyaenids, herpestids, and bovids never colonized South America nor did ursids ever make it to sub-Saharan Africa. But this phylogenetic bias in biogeography should not, in theory, affect the trait-environment relationship in a region because the species that inhabit it are expected to have traits that are compatible with the local environment (11, 15–16). Our focus on two clades (Carnivora and Artiodactyla) means that we sampled a greater proportion of local large mammal communities in North America and Eurasia than we have in South America and Africa. Statistical power of ecometric analyses globally, and in these two continents especially, would be improved by sampling all large carnivores and herbivores, which may include members of clades like Perissodactyla, Xenarthra, or Metatheria.

In much of the northern hemisphere (Fig. 1 *D* and *E*, gray areas), we do not include many sampled communities because the lack of species within the faunal communities (i.e., there are fewer than three artiodactyl or carnivoran species present) means that we cannot evaluate potential mismatch. Today, the artiodactyl fauna of much of eastern North America is limited to only white-tailed deer (*Odocoileus virginianus*), though it was once home to the now extirpated elk (*Cervus elaphus*) (88) and bison (*Bison bison*) (89). Since the arrival of humans, large mammals shifted geographically and climatically into areas with less human presence (43, 90), leaving behind areas depauperate in fauna. In these faunally depauperate areas, we were unable to calculate variance associated with community-level trait values, so we did not include these communities in our models. We were also unable to determine vectors of change for two paleontological sites that occur in these depauperate areas. This breakdown of community structure indicates an overall shift in ecosystem function and highlights areas that could benefit from conservation restoration efforts.

## Conclusions

Our results show that there is an overall strong relationship between trait distributions and vegetation cover both today and in the past and that relationship is better detected as more trophic levels are included in an ecometric likelihood framework. We show that the trait-environment relationship between large mammal locomotion and vegetation cover is relatively consistent through geographic space. Ongoing community reassembly and functional diversity loss are expected to contribute to ecosystem disruption (42, 91, 92). Metrics like the ecometric anomaly may be useful additions to the conservation toolkit. Because ecometric anomalies focus on the functional relationship of animals to their environments, they can be used to identify areas that could be prioritized for conservation management. This approach also captures incompatibilities and adds to the information provided by commonly used indices like species richness or extirpation. Importantly, knowledge of how traits will respond mechanistically to environmental change may provide a more nuanced basis for assessing which species will thrive and which will be threatened [*sensu lato* anticipatory management (93, 94)]. An improved understanding of functional trait dynamics can help inform conservation strategies, whether they be urban–wildlife coexistence programs to support biodiversity in areas of high human influence (95) or constructing connectivity networks to allow movement of animals between protected areas (96, 97), either of which is likely to be more successful if the relevant environment–trait interactions are well understood so that the solution matches the functional properties of the threatened species, communities, and ecosystems.

## Materials and Methods

We integrated species range maps, functional trait measures of locomotor strategy, global vegetation cover, and trait measures from selected fossil localities to evaluate ecometric models for communities of species within the orders of Artiodactyla and Carnivora. All analyses were performed in the R Computing Environment (v4.2.1) (98) and are available on Figshare (99).

### Spatial Community Composition.

Modern, global species range maps were sourced from the International Union for Conservation of Nature (IUCN) Red List Spatial Data (100), downloaded on August 6, 2021. We revised the taxonomy as required to correspond with Mammal Species of the World (101) so that geographic range maps and measured traits could be correctly related. We spatially overlapped range maps and extracted lists of species to approximate community composition for sampling 50-km equidistant points across the globe (n = 54,090 points) following previous ecometric sampling schemes (14, 47, 48, 84). All sampling points were limited to those with species considered extant (presence = 1) and native or reintroduced (origin = 1 or 2) by the IUCN. Previous work found that community trait values are not sensitive to changes in richness, except at the lowest richness levels (48, 82), so we removed sampling point locations with fewer than three species (*SI Appendix*, Table S8).

At each sample point, a species list was produced and used for developing community-level metrics of functional trait distributions. Here, we used the mean and SD of locomotor traits. Although range maps overestimate the community composition and richness at any specific place with species’ ranges (102), these 50-km sampling points offer a way to comparatively sample many points across the globe and approximate the kinds of communities deposited and preserved in the fossil record (47). Alternatively, summarizing communities by either raw occurrence records or by building models of species distributions from those records offers other biases and constraints (103, 104). Abundance data have been shown to improve some ecometric analyses (105), but we could not reliably estimate the abundance for all species of artiodactyls and carnivorans globally.

### Locomotor Efficiency.

We used the ankle gear ratio as an indicator of locomotor efficiency and foot posture. We calculated the ankle gear ratio from two measurements, the overall length of the calcaneum divided by the length of the calcaneal tuber from the proximal end to the sustentacular process (47, 48) (Fig. 1 *A* and *B*). We compiled gear ratio measures from 157 species of artiodactyls (48, 106) and 138 species of carnivorans, including published (16, 47) and previously unpublished data (99). We took the average of the ankle gear ratio measures from multiple specimens to represent an average gear ratio for a species. For each community, we summarized the gear ratio with the mean and SD of its constituent species. We mapped the mean gear ratio to explore functional trait patterns across the globe. We measured the spatial correlation between the community gear ratios of artiodactyls and carnivorans using a Pearson’s correlation. We also modeled the relationship using a second-order polynomial regression after a model selection procedure showed that a simple linear and third-order polynomial regression has less model support than a second-order polynomial regression. Finally, we did not incorporate phylogeny into our ecometric models because, although the traits reflect constrained morphology within clades (23, 47, 48, 84), the ecometric relationship is generally not sensitive to phylogenetic differences across communities (13). Additionally, the efficiency of a species’ ankle gear ratio does not depend on its relatives. It is only dependent on the interaction between its functionality and the local environment (see the *Discussion* for an elaboration).

### Modern Vegetation Cover.

At each 50-km equidistant sampling point, we extracted Matthews’ vegetation cover (59, 60) (*SI Appendix*, Fig. S1). Matthews’ vegetation cover is a global vegetation dataset compiled from published sources and satellite imagery and uses the United Nations Educational, Scientific and Cultural Organization (UNESCO) classification system (59). We chose Matthews’ vegetation cover even though Bailey’s ecoregion has previously explained more trait variance in both taxonomic orders (35, 37). Ecoregion is a variable that combines an interaction of vegetation type, temperature, and precipitation (107). However, temperature and precipitation do not have direct effects on locomotor efficiency, whereas habitat openness and availability of substrates are directly relevant to locomotor efficiency and are largely driven by the type of vegetation cover, so we chose to use a more direct measure of vegetation cover. We simplified Matthews’ vegetation cover categories from 32 to five categories: arctic, deciduous, desert, evergreen, and grassland following Short and Lawing (48) (see *SI Appendix*, Table S9 for the original and simplified categories). Simplified categories allow us to better compare disparate regions across the globe and would allow for a comparison with paleontological estimates of vegetation cover.

### Ecometric Trait-Environment Relationships.

We used likelihood-based ecometric models to assess the relationship between traits and vegetation cover. Likelihood methods were used because they have been shown to produce more accurate estimates from ecometric models than linear regression, polynomial regression, or nearest neighbor (87). With community trait and vegetation data, we built three ecometric models: a) carnivorans only (n = 48,273 communities), b) artiodactyls only (n = 21,062 communities), and c) both (n = 21,056 communities). We refer to the “both” model as the trophically integrated model. At each geographic sampling point, community-level means and SDs of gear ratio were calculated for each taxonomic order. Community-level trait values were then binned into a 25 × 25 matrix grid to produce an ecometric trait space (14, 48, 84).

The carnivoran-only and artiodactyls-only ecometric trait spaces are two-dimensional spaces with mean community values on the x-axis and SD community values on the y-axis. The trophically integrated ecometric trait space is a four-dimensional hypercube made up of four trait measures: artiodactyl mean, artiodactyl SD, carnivoran mean, and carnivoran SD. In this case, each axis was binned into 25 equal units. Those hypercubes were plotted in 2-D space for visualization with means of each group on the x- and y-axes. For each of the ecometric trait bins within each of the ecometric spaces, we calculated the most likely vegetation cover by taking the mode of vegetation cover for all the communities that occur within the bin (48, 87). We also calculated the likelihood for each vegetation cover within a trait bin given the vegetation cover for all the communities within the bin. Ecometric spaces are visual representations of these likelihood models that relate traits to their environments.

To assess the ability of the model to predict vegetation cover with lower sample sizes, we performed a jackknife procedure that randomly resampled the communities at sample sizes ranging from 100 to 12,100 communities by intervals of 400. The random resample for each interval was repeated 20 times. For each iteration and sample size, we randomly selected 80% of the data to train an ecometric model, and we used the remaining 20% to test the model. We evaluated the ability of the training and testing data to predict the observed vegetation cover by taking the percent of communities with anomalies lower than 0.3. We chose 0.3 because communities with such low anomalies indicate that the observed vegetation was either correct or nearly as likely as the most likely vegetation. We found that the accuracy of the training data decreases with increased sample size and stabilizes (*SI Appendix*, Fig. S9). The percent of communities with anomalies less than 0.3 increased to approximately 75% around a sample size of 6,000, indicating that the much larger sample size of the full model is adequate to capture the trait-environment relationship.

### Ecometric Anomalies.

To evaluate the ecometric models, we compared the observed vegetation cover to the vegetation cover estimated by each model. For each community, trait values were placed in ecometric space, and a vegetation estimate was calculated based on the trait bin. A previous analysis of randomly partitioning data into training and testing data showed that this is a reliable method for evaluating the accuracy of the ecometric models (48). From the model estimates of vegetation cover, we calculated three measures by assessing whether the trait-based vegetation estimates matched Matthews’ original vegetation cover: a) a binary (yes or no) count of how many 50-km sampling points had estimated vegetation that was correct with respect to observed vegetation (14, 48, 87), b) a Cohen’s kappa (κ) score that adjusts for the correct classification arising by chance and yields a score between 0 (no agreement greater than chance) and 1 (perfect agreement) (15, 61, 62), and c) an ecometric anomaly, the maximum likelihood minus the likelihood of the observed vegetation type. Cohen’s kappa provides a summary of the proportion of correct classification that is corrected for chance agreement between modeled and observed vegetation. The ecometric anomaly enables us to investigate patterns within the incorrect estimates. For this measure, we calculate the difference between two likelihoods in each community: the maximum likelihood of the estimated vegetation and the likelihood of the observed vegetation type. The ecometric anomalies range between 0 and 1, with the anomalies that are zero equivalent to the correct correctly classified vegetation types. For the incorrect matches, the higher the ecometric anomaly, the greater the difference between the maximum likelihood and the likelihood of the observed vegetation.

### Paleontological Sites.

Paleontological sites were sourced from the Neotoma database and restricted by age to limit the amount of time-averaging at our study sites (maximum site age greater than 999 y ago and less than 120,001 y ago, and site age range less than 5,000 y) (n = 1,040 sites). We chose these parameters so that we removed sites that were entirely within the past 1,000 y to minimize the role of increasing human presence on community composition, those that were older than 120,000 to minimize the occurrence of Pleistocene taxa and those for which time averaging may have been too great to be representative of the assemblage. We selected sites that included three or more artiodactyl species and three or more carnivoran species (n = 46). Older sites could have been included if trait data were available for the taxa occurring within those sites. Stratigraphic units within a site were lumped because together they had enough taxa. Five of those sites for which a paleovegetation estimate was obtained from the integrated ecometric model were selected for our example (*SI Appendix*, Table S5).

Species lists were obtained for each site and used to calculate trait means and SDs for each community. Sites were placed in ecometric space, and paleovegetation was estimated from the trophically integrated model. We compiled independent published interpretations of the paleoenvironment for each of the sites and compared those with ecometric reconstructions (*SI Appendix*, Table S6). The nearest 50-km sampling point from our modern ecometric models was identified to represent a corresponding modern community at a coincident geographic location. Modern and past fauna were compared using trait means and SDs to evaluate functional trait turnover through time (*SI Appendix*, Table S7). In doing so, we plotted vectors of trait change in an ecometric space.

## Supplementary Material

Appendix 01 (PDF)

## Data Availability

Code and data have been deposited at https://doi.org/10.6084/m9.figshare.19358513.v1 (99).
