# Supplementary material for: Trophically integrated ecometric models as tools for demonstrating spatial and temporal functional changes in mammal communities
Source: Proc Natl Acad Sci U S A. 2023 Feb 6;120(7):e2201947120. doi: 10.1073/pnas.2201947120 (PMC9963252; doi:10.1073/pnas.2201947120)
Supplement: Supplementary file 1 — Appendix 01 (PDF) [file pnas.2201947120.sapp.pdf]

PNAS
www.pnas.org

Supplementary Information for

Trophically integrated ecometric models as tools for demonstrating spatial and temporal functional changes in mammal communities

Rachel A. Short[1,2,3*], Jenny L. McGuire[3,4,5], P. David Polly[6], A. Michelle Lawing[2*]

[1]Department of Natural Resource Management, South Dakota State University, Rapid City, SD

[2]Department of Ecology and Conservation Biology, Texas A&M University, College Station, TX

[3]School of Biological Sciences, Georgia Institute of Technology, Atlanta, GA

[4]School of Earth and Atmospheric Sciences, Georgia Institute of Technology, Atlanta, GA

[5]Interdisciplinary Graduate Program in Quantitative Biosciences, Georgia Institute of Technology, Atlanta, GA

[6]Department of Earth and Atmospheric Sciences, Indiana University, Bloomington, IN

*Rachel A. Short and A. Michelle Lawing

Email: rachel.short@sdstate.edu, alawing@tamu.edu

This PDF file includes:

Figures S1 to S9

Tables S1 to S9

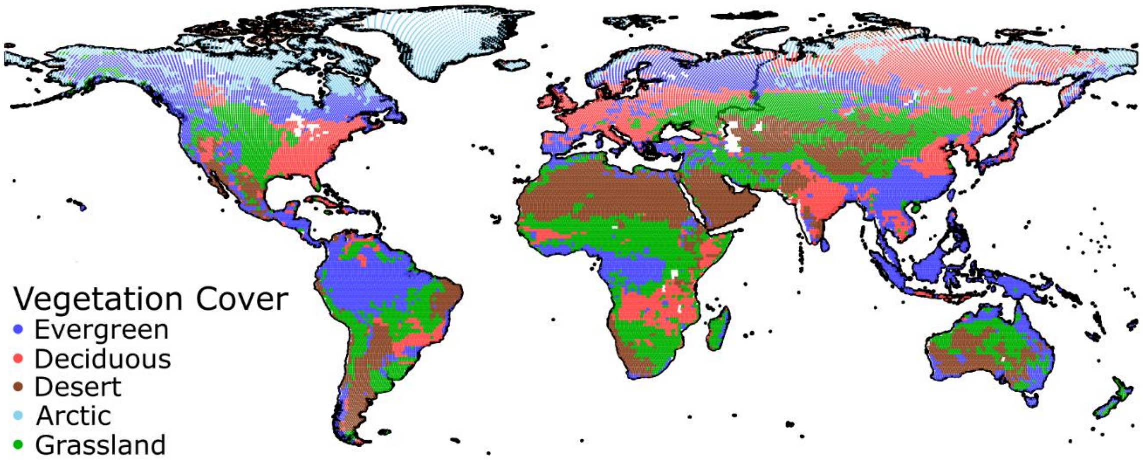

Figure S1. Vegetation cover simplified from Matthews' vegetation cover (1). The full and simplified vegetation categories are listed in Table S9.

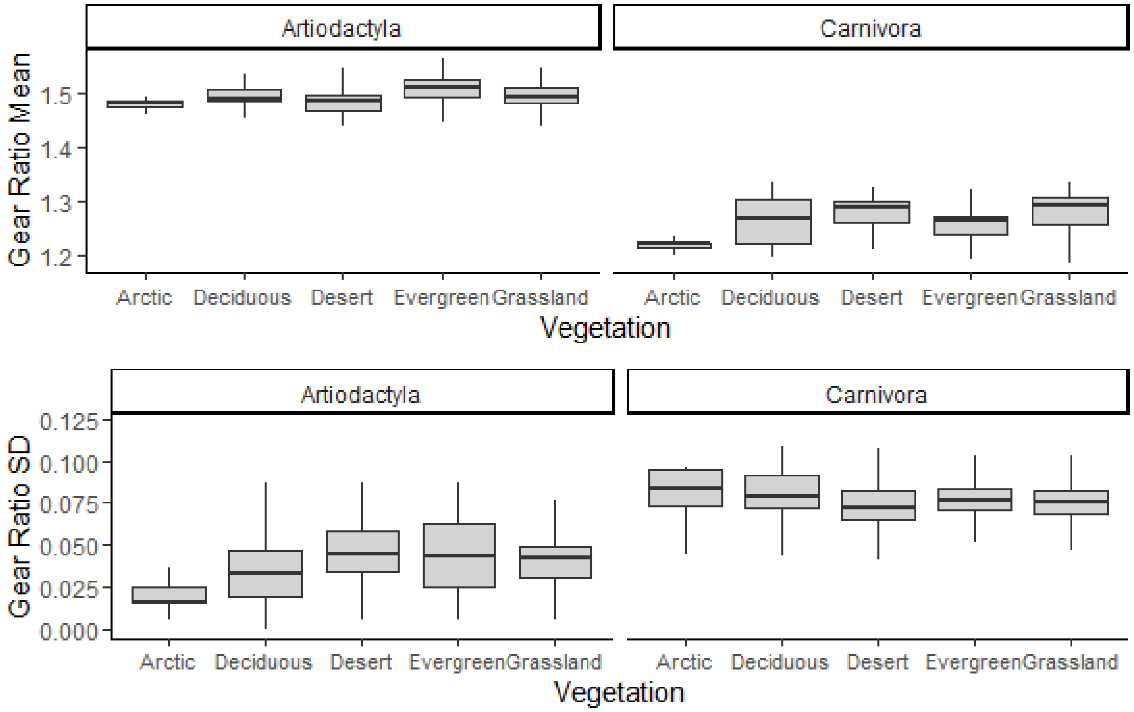

Figure S2. Distribution of mean and standard deviation of gear ratios in each vegetation type for Artiodactyla and Carnivora communities.

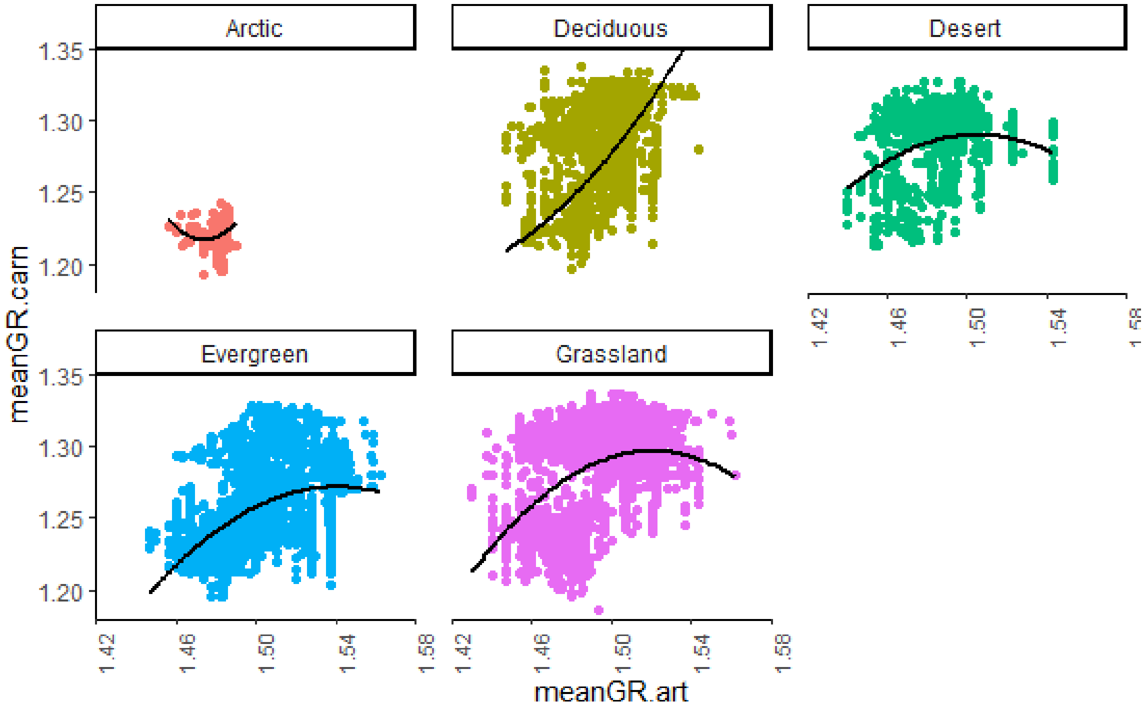

Figure S3. Distribution of means of gear ratios within each vegetation type for Artiodactyla and Carnivora. The second order polynomial curves are plotted for each vegetation type. Arctic (n = 374), Deciduous (n = 5215), Desert (n = 1538), Evergreen (n = 7569) and Grassland (n = 6067). Each point is mean gear ratio summarized for each community from our systematic sampling at 50 km equidistant points across the globe.

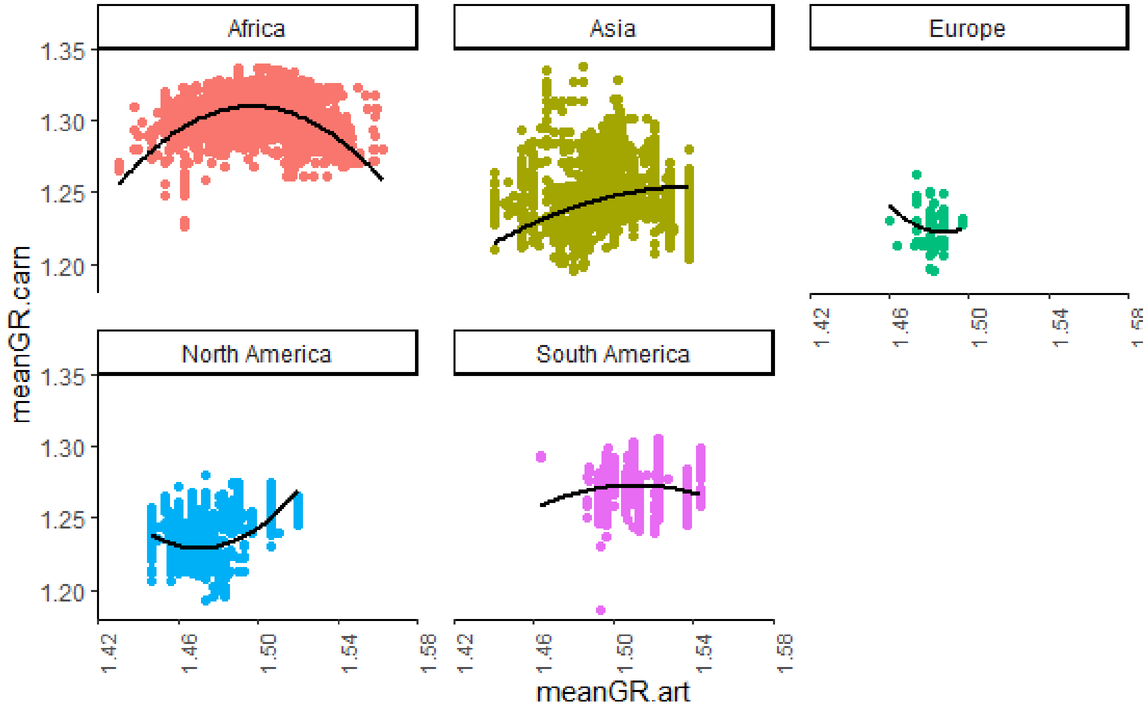

Figure S4. Distribution of means of gear ratios within each continent for Artiodactyla and Carnivora. The second order polynomial curves are plotted for each continent. Africa (n = 7307), Asia (n = 6271), Europe (n = 530), North America (n = 2494) and South America (n = 4454). Each point is mean gear ratio summarized for each community from our systematic sampling at 50 km equidistant points across the globe.

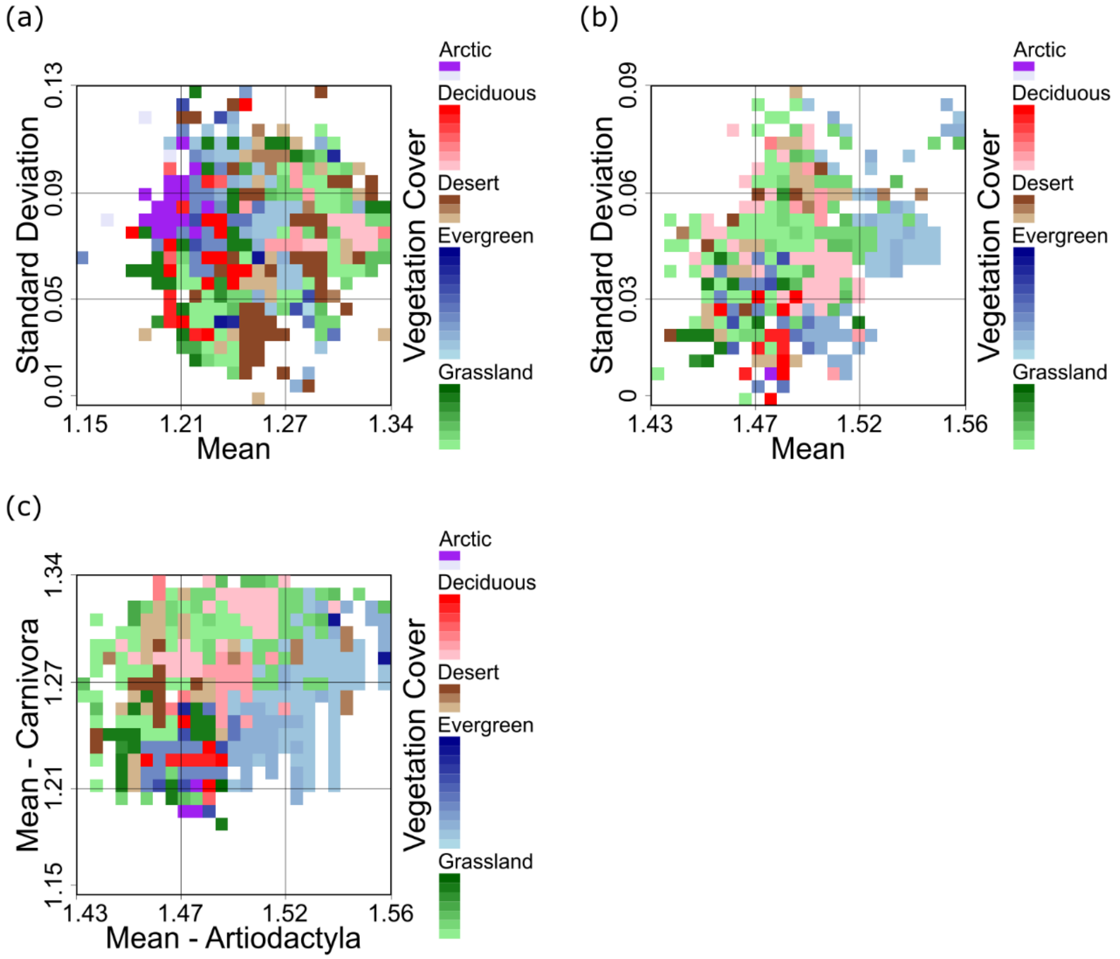

Figure S5. Ecometric spaces showing the most likely vegetation cover given the mean and standard deviation of gear ratios that occur within each ecometric bin. (a) carnivorans only; (b) artiodactyls only; (c) trophically integrated.

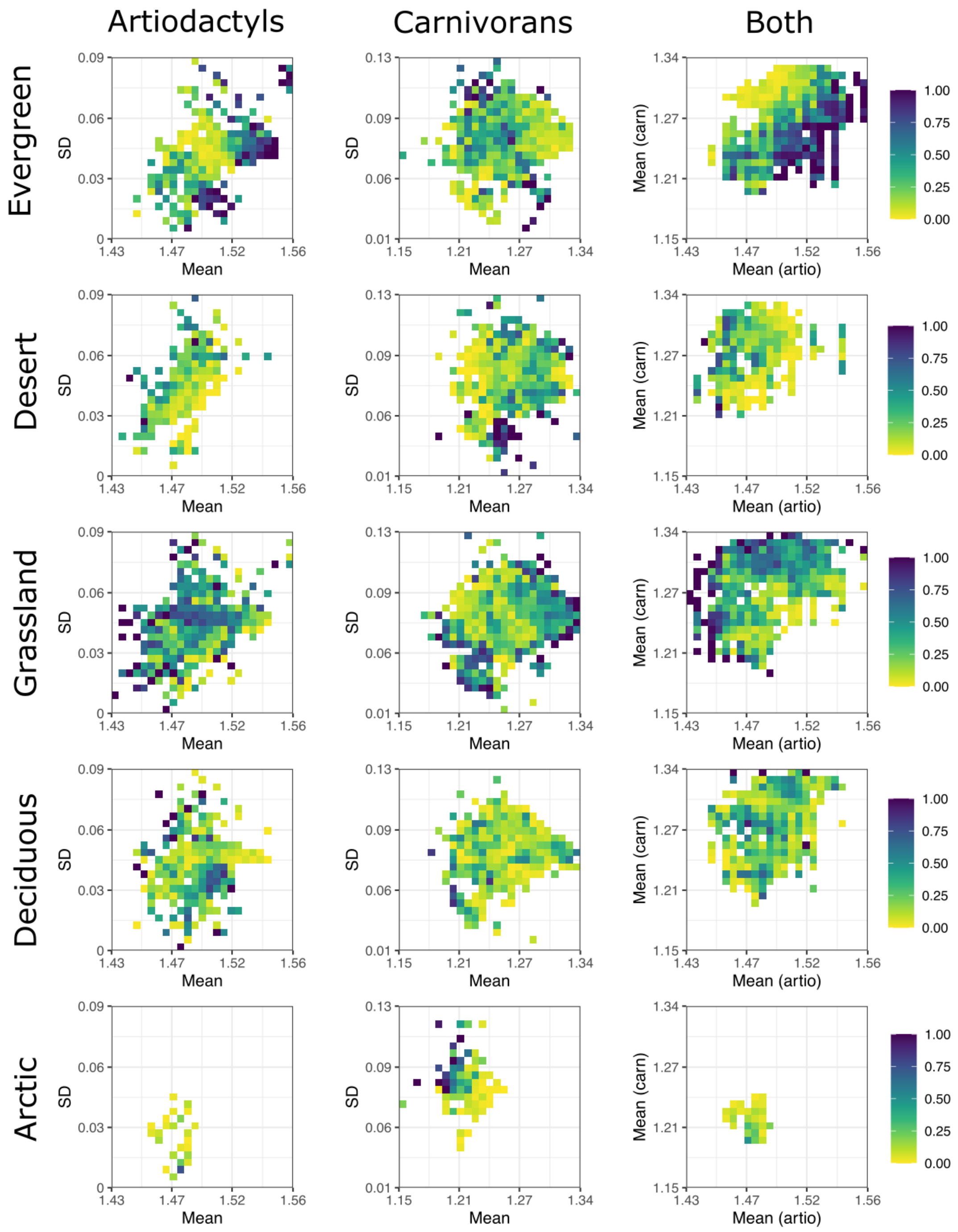

Figure S6. Likelihood surfaces of the ecometric trait spaces for Artiodactyla (first column), Carnivora (second column), and the integrated model (third column) for each of five simplified vegetation categories (rows). The ecometric trait spaces for the first two columns show mean on the x axis and standard deviation on the y axis. The third column of spaces shows the artiodactyl mean on the x axis and the carnivoran mean on the y axis. The color gradients represent the likelihood for each vegetation type given the mean and standard deviation of the gear ratios that occur within each ecometric trait bin.

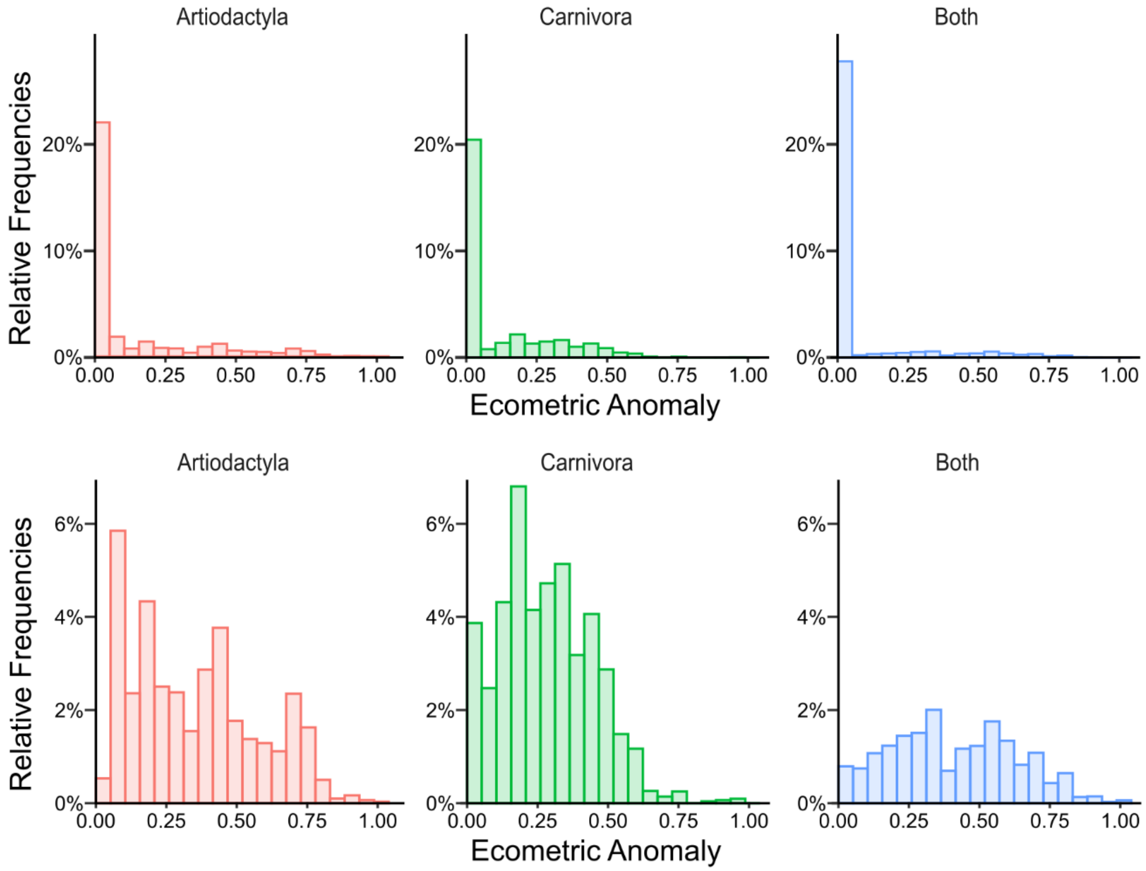

Figure S7. Distribution of ecometric anomalies for each of the three ecometric models (Artiodactyla only, Carnivora only and trophically integrated). The top row includes communities that had correctly classified vegetation types from the ecometric models, where the ecometric anomaly equals zero, and communities that do not have correctly classified vegetation type, where the ecometric anomaly is greater than zero. The bottom row displays only the communities that do not have correctly classified vegetation type, where the ecometric anomaly is greater than zero.

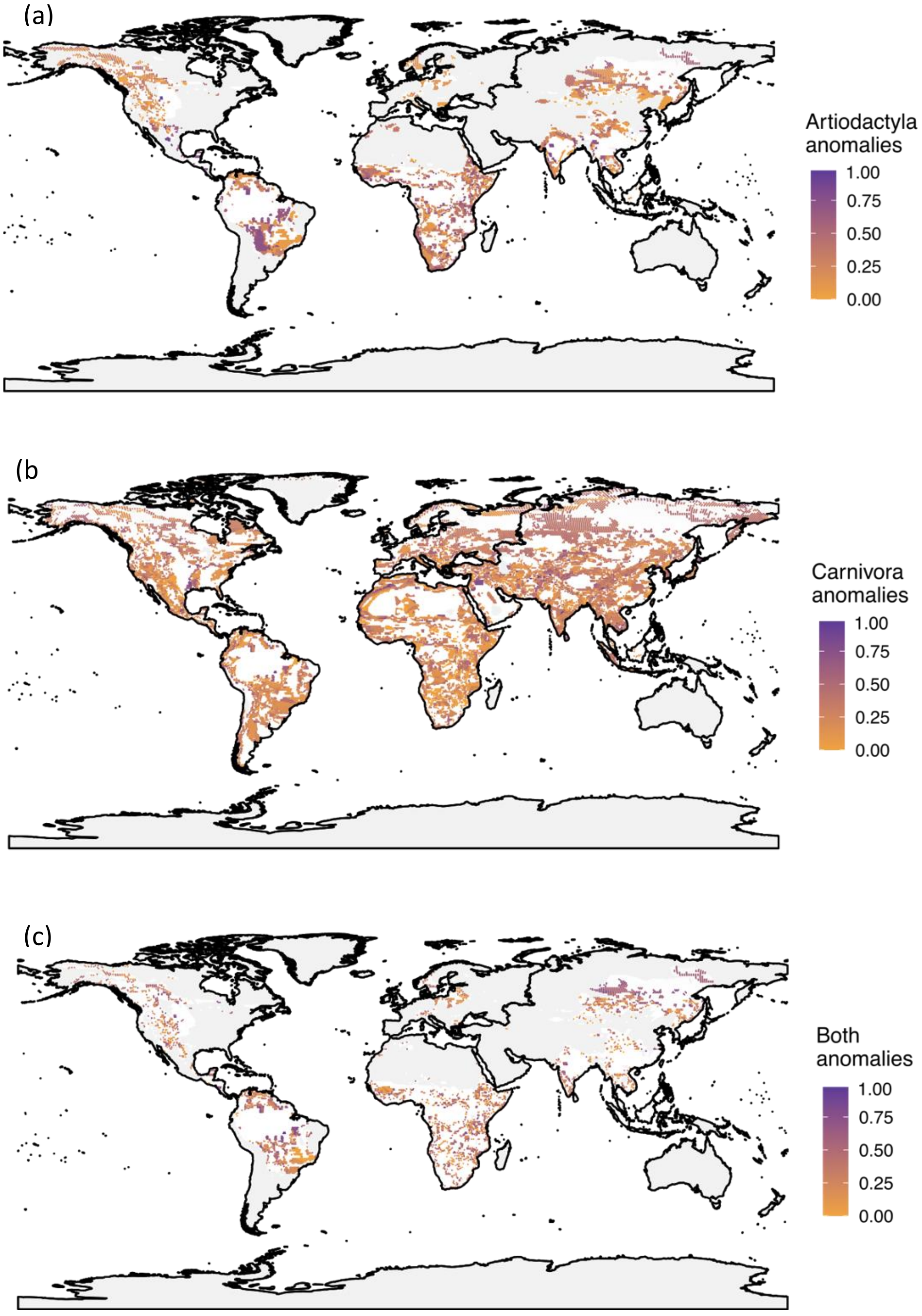

Figure S8. Geographic distribution of the ecometric anomalies for (a) the Artiodactyla model, (b) the Carnivora model, and (c) the trophically integrated model. Anomalies are calculated from the likelihood value of the most likely vegetation type minus the likelihood value of the observed vegetation type given observed gear ratios within ecometric trait bins. An ecometric anomaly of zero is white and indicates that the most likely vegetation type is also the observed vegetation type. There are more communities with white or lighter hues in the trophically integrated model.

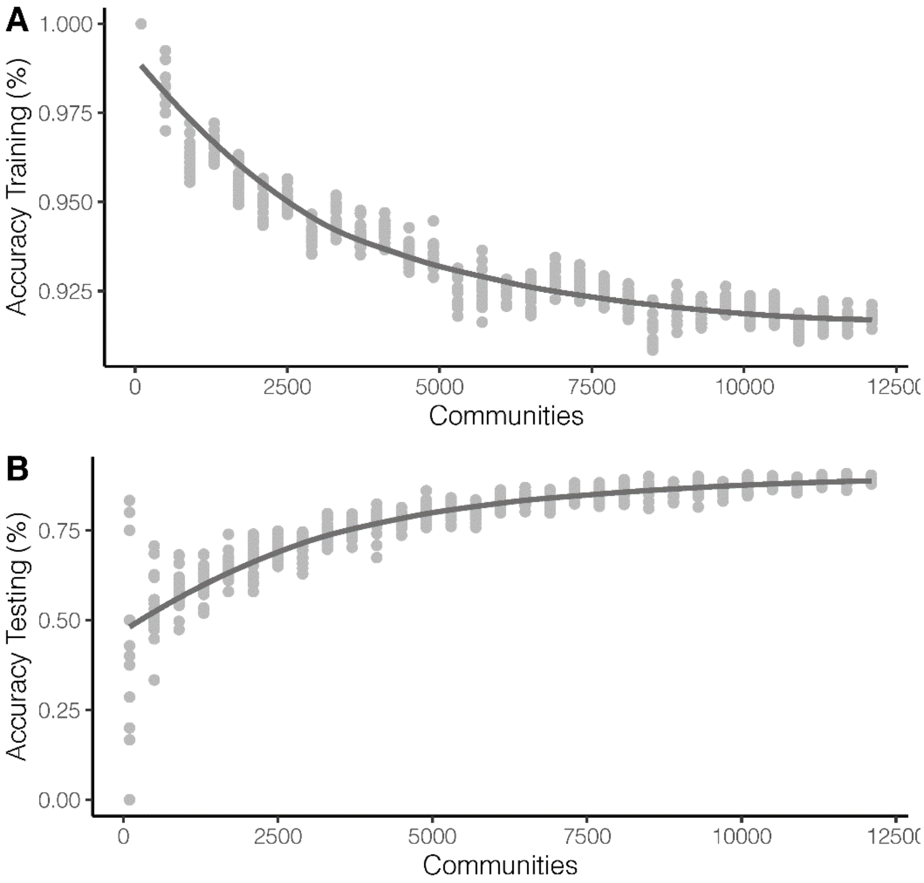

Figure S9. Accuracy of training data (a) and testing data (b) measured by the percent of communities that have ecometric anomalies less than 0.3. The loess curve is fitted to the data using default parameters for the loess() function in R with default parameters.

Table S1. Analysis of variance table evaluating the gear ratio across vegetation types for each community level metric (artiodactyl mean $R^2_{adj} = 0.16$, $p < 0.01$, artiodactyl standard deviation $R^2_{adj} = 0.04$, $p < 0.01$, carnivoran mean $R^2_{adj} = 0.14$, $p < 0.01$, and carnivoran standard deviation $R^2_{adj} = 0.07$, $p < 0.01$).

	Df	Sum Sq	Mean Sq	F value	Pr (>F)
**Artiodactyl mean**					
Vegetation type	4	1.64	0.409	957.75	< 2.2e-16
Residuals	20758	8.87	0.0004		
**Artiodactyl standard deviation**					
Vegetation type	4	0.12	0.0293	196.68	< 2.2e-16
Residuals	20758	3.09	0.0001		
**Carnivoran mean**					
Vegetation type	4	3.16	0.789	823.31	< 2.2e-16
Residuals	20758	19.91	0.01		
**Carnivoran standard deviation**					
Vegetation type	4	0.511	0.128	391.7	< 2.2e-16
Residuals	20758	6.77	0.0003		

Table S2. Gear ratio data for each continent. N values refer to the number of communities in the integrated model. Mean, standard deviation, minimum, maximum, and range are provided for the mean gear ratio and the standard deviation of gear ratio for both trophic levels.

Continent	Model	Mean	Standard Deviation	Minimum	Maximum	Range
Africa (n = 7307)						
	Mean Artiodactyla	1.50	0.02	1.43	1.56	0.13
	SD Artiodactyla	0.04	0.01	0.00	0.08	0.08
	Mean Carnivora	1.30	0.02	1.23	1.34	0.11
	SD Carnivora	0.07	0.01	0.03	0.11	0.08
Asia (n = 6271)						
	Mean Artiodactyla	1.49	0.02	1.44	1.54	0.10
	SD Artiodactyla	0.03	0.01	0.01	0.06	0.05
	Mean Carnivora	1.24	0.02	1.20	1.34	0.14
	SD Carnivora	0.08	0.01	0.02	0.12	0.10
Europe (n = 530)						
	Mean Artiodactyla	1.48	0.00	1.46	1.50	0.04
	SD Artiodactyla	0.01	0.01	0.00	0.03	0.03
	Mean Carnivora	1.22	0.01	1.20	1.26	0.07
	SD Carnivora	0.06	0.02	0.03	0.09	0.06
North America (n = 2494)						
	Mean Artiodactyla	1.47	0.02	1.45	1.52	0.07
	SD Artiodactyla	0.04	0.02	0.01	0.09	0.08
	Mean Carnivora	1.23	0.02	1.19	1.28	0.09
	SD Carnivora	0.07	0.01	0.03	0.10	0.07
South America (n = 4454)						
	Mean Artiodactyla	1.52	0.01	1.46	1.54	0.08
	SD Artiodactyla	0.06	0.00	0.02	0.08	0.07
	Mean Carnivora	1.27	0.01	1.19	1.31	0.12
	SD Carnivora	0.08	0.01	0.07	0.11	0.04

Table S3. Linear mixed effects model evaluating the relationship between mean gear ratios of communities of artiodactyls and carnivorans with the random effect of continent. The correlation of fixed effects for this model is -0.553.

Fixed Effects					
	numDf	denDF	Coefficient	F value	Pr (>F)
Intercept	1	21050	1.13	7821.20	< 0.0001
Artiodactyla Gear Ratio	1	21050	0.08	167.80	< 0.0001
Random Effect					
	Observations	Variance	Std Dev		
Continent	5	0.0101	0.0317		
Residual	21056	0.00029	0.0169		

Table S4. Agreement between the estimated vegetation and observed vegetation for each model (carnivoran only, n = 47,270, artiodactyl only, n = 20,766, and trophically integrated, n = 20,763) and within each vegetation type. Kappa scores were only calculated for the complete dataset of vegetation cover.

Vegetation	Carnivora			Artiodactyla			Integrated		
	Agreement	Kappa	p value	Agreement	Kappa	p value	Agreement	Kappa	p value
All	57.66%	0.45	0	64.75%	0.50	0	80.85%	0.73	0
Arctic	56.96%	-	-	5.61%	-	-	22.19%	-	-
Deciduous	47.35%	-	-	62.30%	-	-	77.66%	-	-
Desert	60.62%	-	-	26.72%	-	-	77.24%	-	-
Evergreen	62.01%	-	-	75.33%	-	-	87.24%	-	-
Grassland	59.49%	-	-	66.94%	-	-	80.14%	-	-

Table S5. Select paleontological sites. Site ID is an identifier from the Neotoma Database (2). Richness and trait values are calculated from species lists housed in the Neotoma Database.

	Site Name	Site ID	Age		Artiodactyl			Carnivoran		
			Minimum	Maximum	Richness	Mean gear ratio	SD gear ratio	Richness	Mean gear ratio	SD gear ratio
1.	Sjovold [EiNs-4]	23638	0	4500	3	1.483	0.0208	6	1.232	0.0893
2.	Lamar	4367	1	1695	4	1.490	0.0258	6	1.218	0.0703
3.	Bear River No. 3	4980	950	1500	3	1.473	0.0416	5	1.230	0.0892
4.	Fisher	5763	550	1650	3	1.487	0.0416	11	1.238	0.0590
5.	McKinstry [21KC2]	5893	1150	1650	4	1.480	0.0337	10	1.230	0.0897

Table S6. Modern and past vegetation types at select paleontological sites. Estimated vegetation types are reported from the trophically integrated model. When two vegetation types had high probabilities, we have provided both. The modern estimated and modern observed vegetation types are reported from the nearest modern sampling point to each paleontological site, except for Fisher and McKinstry sites because they are too far from any of our modern sampling points. Modern observed data are from our simplified version of Matthews' vegetation cover (see Table S9). The paleoenvironmental interpretation is also simplified from discussions in the literature, cited in the References.

	Site Name	Site ID	Vegetation				References
			Modern Estimated	Modern Observed	Paleo Estimated	Paleo Interpretation	
1.	Sjovold [EiNs-4]	23638	grassland	grassland	deciduous/evergreen	Grasslands to parklands transition	(3–7)
2.	Lamar	4367	evergreen	evergreen	evergreen	Mixed evergreen forest and grasslands, Forest habitats (evergreen) persisted from then to now in same relative abundance, Dense tall grass habitats reduced to sparse and arid grasslands habitats	(8, 9)
3.	Bear River No. 3	4980	grassland	grassland	deciduous	Grassland with increasing mosaic habitats towards modern that decreased grassland connectivity	(10–12)
4.	Fisher	5763	NA	NA	evergreen/grassland	"Tundra forest" to boreal forest	(13–15)
5.	McKinstry [21KC2]	5893	NA	NA	deciduous	Mixed forest (evergreens and deciduous)	(16, 17)

Table S7. Fauna recorded at each site in the fossil community and in the modern community. Site ID is an identifier from the Neotoma Database (2). Point ID is the nearest sampling point from which the modern communities were extracted. O ID is the species list compiled from sampling range maps at the Point ID.

	Site Name	Bear River No. 3		Lamar		Sjovold [EiNs-4]	
	Site ID	4980		4367		23638	
	Point ID	136819		132163		124709	
	O ID	32141		29827		25600	
Order	Species	Time Period					
		Fossil	Modern	Fossil	Modern	Fossil	Modern
Artiodactyla	*Alces americanus*					x	
	Antilocapra americana	x	x	x		x	x
	Bison bison	x		x			
	Cervus elaphus		x	x	x	x	
	Odocoileus hemionus	x	x		x		x
	Odocoileus virginianus		x		x		x
	Ovis canadensis			x			
Carnivora	*Canis latrans*		x	x	x		x
	Canis lupus			x	x	x	
	Lontra canadensis	x	x		x		x
	Lynx canadensis					x	
	Lynx rufus	x	x		x		
	Martes pennanti					x	
	Mephitis mephitis	x	x	x	x		x
	Mustela erminea		x		x		
	Mustela frenata		x		x		x
	Mustela nivalis						x
	Neovison vison	x			x		x
	Procyon lotor				x		x
	Puma concolor		x		x		
	Spilogale gracilis		x				
	Taxidea taxus		x	x	x	x	x
	Urocyon cinereoargenteus		x				
	Ursus americanus	x			x		
	Ursus arctos			x	x	x	
	Vulpes velox					x	
	Vulpes vulpes			x			

Table S8. Sample sizes of communities associated with the systematic 50 km equidistant sampling scheme for carnivoran and artiodactyl communities within and among regions.

		Richness > 0		Richness > 2	
Regions	All	Carnivora	Artiodactyla	Carnivora	Artiodactyla
Global	54090	49838	47404	48682	25659
Africa	12046	11968	11653	11835	7355
Asia	17988	17351	16651	17148	9605
Europe	3961	3913	3722	3802	1641
North America	9699	9488	8682	8905	2504
South America	7132	7110	6694	6992	4554

Table S9. Corresponding vegetation cover categories between Matthews' vegetation cover (1) and the simplified version from Short and Lawing (18).

Matthews' Vegetation Number	Matthews' Vegetation Name	Simplified Vegetation Number	Simplified Vegetation Name
1	tropical evergreen rainforest	1	evergreen
2	trop/subtropical evergreen seasonal broad-leaved forest	1	evergreen
3	subtropical evergreen rainforest	1	evergreen
4	temperate/subpolar evergreen rainforest	1	evergreen
5	temperate evergreen seasonal broadleaved forest, summer rain	1	evergreen
6	evergreen broadleaved sclerophyllous forest, winter rain	1	evergreen
7	tropical/subtropical evergreen needle-leaved forest	1	evergreen
8	temperate/subpolar evergreen needle-leaved forest	1	evergreen
9	tropical/subtropical drought-deciduous forest	2	deciduous
10	cold-deciduous forest, with evergreens	2	deciduous
11	cold-deciduous forest, without evergreens	2	deciduous
12	xeromorphic forest/woodland	3	desert
13	evergreen broadleaved sclerophyllous woodland	1	evergreen
14	evergreen needle-leaved woodland	1	evergreen
15	tropical/subtropical drought-deciduous woodland	2	deciduous
16	cold-deciduous woodland	2	deciduous
17	evergreen broadleaved shrubland/thick, evergreen dwarf-shrubland	1	evergreen
18	evergreen needle-leaved or microphyllous shrubland/thicket	1	evergreen
19	drought-deciduous shrubland/thicket	2	deciduous
20	cold-deciduous subalpine/subpolar shrubland/dwarf shrub	2	deciduous
21	xeromorphic shrubland/dwarf shrubland	3	desert
22	arctic/alpine tundra, mossy bog	4	arctic
23	tall/medium/short grassland, 10-40% woody cover	5	grassland

24	tall/medium/short grassland, < 10% woody cover	5	grassland
25	tall/medium/short grassland, shrub cover	5	grassland
26	tall grassland, no woody cover	5	grassland
27	medium grassland, no woody cover	5	grassland
28	meadow, short grassland, no woody cover	5	grassland
29	forb formations	5	grassland
30	desert	3	desert
31	ice	4	arctic
32	cultivation	NA	NA

SI References

1. E. Matthews, Global Vegetation Types, 1971-1982 (Matthews). Data set. (1999) https:/doi.org/10.3334/ORNLDAAC/419.

2. J. W. Williams, *et al.*, The Neotoma Paleoecology Database, a multiproxy, international, community-curated data resource. *Quat. Res. (United States)* **89**, 156–177 (2018).

3. I. Dyck, R. E. Morlan, *The Sjovold Site: A river crossing campground in the Northern Plains* (University of Ottawa Press, 1995).

4. I. G. Dyck, “The prehistory of southern Saskatchewan” in *Tracking Ancient Hunters: Prehistoric Archaeology in Saskatchewan*, H. T. Epp, I. G. Dyck, Eds. (Saskatchewan Archaeological Society, 1983), pp. 63–139.

5. R. E. Morlan, R. McNeely, S. A. Wolfe, B. T. Schreiner, Quaternary dates and vertebrate faunas in Saskatchewan. *Open File Report, Geol. Surv. Canada.* **3888**, 1–139 (2002).

6. R. E. Morlan, A compilation and evaluation of radiocarbon dates in Saskatchewan. *Saskatchewan Archaeol.* **13**, 3–84 (1993).

7. A. A. Rutherford, J. Wittenberg, B. C. Gordon, University of Saskatchewan radiocarbon dates X. *Radiocarbon* **26**, 241–292 (1984).

8. E. A. Hadly, “Late Holocene mammalian fauna of Lamar Cave and its implications for ecosystem dynamics in Yellowstone National Park, Wyoming,” Northern Arizona University. (1990).

9. E. H. Barnosky, Ecosystem dynamics through the past 2000 years as revealed by fossil mammals from Lamar Cave in Yellowstone National Park, USA. *Hist. Biol.* **8**, 71–90 (1994).

10. D. B. Madsen, “The human prehistory of the Great Salt Lake Region” in *Great Salt Lake: A Scientific, Historical, and Economic Overview*, J. W. Gwynn, Ed. (Utah Geological and Mineral Survey Bulletin 116, 1980), pp. 19–32.

11. W. F. Shields, G. F. Dalley, The Beaver River No. 3 site. *Univ. Utah, Anthropol. Pap.* **99**, 55–99 (1978).

12. K. D. Lupo, D. N. Schmitt, On late Holocene variability in bison populations in the northeastern Great Basin. *J. Calif. Gt. Basin Anthropol.* **19**, 50–69 (1997).

13. P. W. Parmalee, The faunal complex of the Fisher site, Illinois. *Am. Midl. Nat.* **68**, 399–408 (1962).

14. P. F. Karrow, T. W. Anderson, A. H. Clarke, L. D. Delorme, M. R. Sreenivasa, Stratigraphy, paleontology, and age of Lake Algonquin sediments in southwestern Ontario, Canada. *Quat. Res.* **5**, 49–87 (1975).

15. P. L. Storck, The Fisher site: Archaeological, geological, and paleobotanical studies at an Early Paleo-Indian site in southern Ontario, Canada. *Mem. Museum Anthropol.* **30**, 1–327 (1997).

16. P. W. J. Lukens, “Some ethnozoological implications of mammalian faunas from Minnesota archeological sites,” University of Minnesota. (1963).

17. J. Richner, “Expressions of the Past: Archeological Research at Voyageurs National Park”

(National Park Service, 2008).

18. R. A. Short, A. M. Lawing, Geography of artiodactyl locomotor morphology as an environmental predictor. *Divers. Distrib.* **27**, 1818–1831 (2021).
